# Identification of Scams in Initial Coin Offerings With Machine Learning

**DOI:** 10.3389/frai.2021.718450

**Published:** 2021-10-05

**Authors:** Bedil Karimov, Piotr Wójcik

**Affiliations:** Faculty of Economic Sciences, University of Warsaw, Warsaw, Poland

**Keywords:** initial coin offering, fintech, blockchain, crypto, risk management, explainable artificial intelligence

## Abstract

Following the emergence of cryptocurrencies, the field of digital assets experienced a sudden explosion of interest among institutional investors. However, regarding ICOs, there were a lot of scams involving the disappearance of firms after they had collected significant amounts of funds. We study how well one can predict if an offering will turn out to be a scam, doing so based on the characteristics known ex-ante. We therefore examine which of these characteristics are the most important predictors of a scam, and how they influence the probability of a scam. We use detailed data with 160 features from about 300 ICOs that took place before March 2018 and succeeded in raising most of their required capital. Various machine learning algorithms are applied together with novel XAI tools in order to identify the most important predictors of an offering’s failure and understand the shape of relationships. It turns out that based on the features known ex-ante, one can predict a scam with an accuracy of about 65–70%, and that nonlinear machine learning models perform better than traditional logistic regression and its regularized extensions.

## Introduction

**FIGURE 1:**
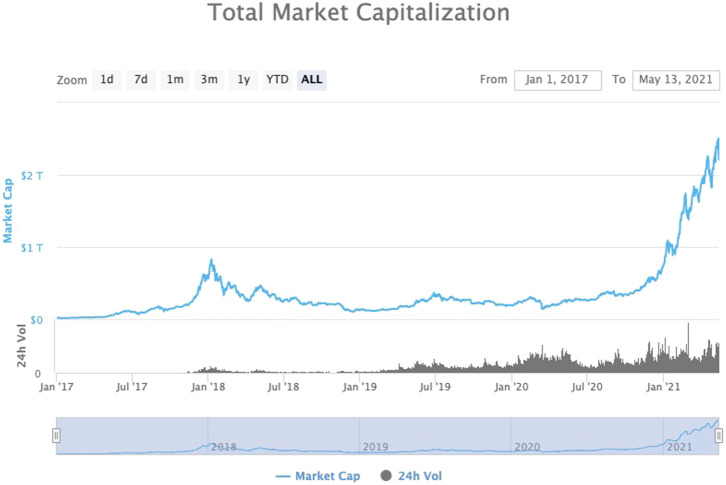
Total market capitalization (log). Source: Coinmarketcap.com.

Over the last 3–4 years, there has been rising interest in the adaptation and use of digital assets like cryptocurrencies (e.g., Bitcoin, Ethereum, Ripple). These products became popular for the first time during the 2017–2018 hype when they reached almost one trillion in capitalization. According to data provider coinmarketcap.com, the peak of the daily trading volume of cryptocurrencies approached the average daily volume of the NYSE in 2017. Nowadays, one may observe a second wave of increasing interest in cryptos, with the market capitalization having reached almost $2.3 trillion in valuation (Figure 1).

Unlike centralized electronic money and central banking systems, most digital tokens are independent of central authorities. The control of these decentralized systems operates within a blockchain, which is an open and distributed ledger that continuously expands. The emergence of cryptocurrencies and coins led to the establishment of new disruptive products like DeFi^1^, NFT^2^, and initial coin offerings (ICOs). Innovative ventures require financial resources to succeed (Gompers and Lerner, 2004). Because of their decentralized nature, the funding of digital assets does not need to go through all the traditional processes, but only through initial coin offerings (ICOs) (Chohan, 2017). In the case of ICOs, new ventures raise funds by selling tokens to a pool of investors. ICOs enable startups to raise large amounts of funding with minimal effort while avoiding compliance and intermediary costs (Kaal and Dell'Erba, 2018). This field is still new and lacks transparency. The amount of objective information surrounding ICOs is very meager, and there is thus considerable potential for fraud (Shifflett and Jones, 2018). Despite the fact that ICOs are able to provide fair and lawful investment opportunities, the ease of crowdfunding creates opportunities and incentives for unscrupulous businesses to use ICOs to execute “pump and dump” schemes, in which ICO initiators drive up the value of the crowdfunded cryptocurrency and then quickly “dump” the coins for a profit (Bian et al., 2018). Due to the high investment risk involved, the US Securities and Exchange Commission (SEC) issued a warning to investors about ICOs, but also acknowledged their innovative potential (OECD, 2019).

Given the previous studies related to this issue, we use data on ICOs to observe which characteristics of an ICO lead to its success or failure. In our case, we include a wide range of control variables that consider additional characteristics of ICOs, with our main focus on features known in advance (ex-ante). In this way, we understand in particular which factors are conducive to success. To achieve this, we employ machine learning algorithms—namely, several linear (e.g., logistic regression and its regularized extensions—LASSO and ridge) and nonlinear (SVM, xgboost, random forest, catboost, and lightgbm) classification algorithms. Given the wide set of variables used, we argue that the nonlinear models better capture the relationships between success and its predictors and better perform classification. Moreover, we consider the technological background of the venture (e.g., the use of blockchain) and the ability to raise funds sufficient for the project’s development to lead to success. Additionally, we use novel, explainable artificial intelligence (XAI) tools to identify the most important predictors of an offering’s failure and to uncover the shape of their relationships with the outcome variable.

The results of our analysis show that extreme gradient boosting (xgboost) is the best algorithm to identify scam ICOs. Additionally, our findings suggest how investors can evaluate and how ventures can conduct a successful ICO. Factors related to ventures’ technological capabilities (e.g., availability of code, use of a decentralized platform in the project) are important determinants of an ICO’s success. This implies that investors should familiarize themselves with DLT and blockchain technology in order to be able to more accurately understand the technical information provided by the ventures (Fisch, 2018). The results also show that the length of crowdsale, the total number of tokens, and the share of tokens kept by the team or offered in a presale to investors are of crucial importance. Grasping the vulnerability of ventures and the importance of these factors reduces the considerable uncertainty and leads to more informed decision-making.

The rest of this study is organized as follows: we give a brief outline of cryptocurrencies, blockchains, and ICO market conditions in *Initial Coin Offerings*. *Predicting the Success of ICOs* presents a review of the literature. In *Research Framework and Methodology*, we explain our methodological framework and methodology, while *Data* describes the dataset of ICO projects and provides some basic analysis of the data. *Empirical Analysis* presents the results of our empirical analysis, and this is followed by our conclusions.

## Initial Coin Offerings

### General Overview

Starting 2016, there was a huge increase in the flow of funds to ventures through a procedure called initial coin offering (ICO), with their amount increasing from 39 total up until 2016 to 256 in 2016 alone (Stanley, 2019). ICO companies from various industries raise funds to develop their product itself or the company’s position on the market. Basically, the idea of ICOs is the same as crowdfunding as both methods allow startups and entrepreneurs to raise funds for their project through the Internet, outside of traditional financing channels (Ante et al., 2018; Lee and Parlour, 2021). The funds that are raised during ICOs are basically for projects that are at concept level, whereas with crowdfunding, the project is at a certain stage. However, there are a few nuances—like white papers^3^ and the creation of tokens—that are not applied in crowdfunding. In this process, the company first has to come up with a product, which may involve the usage of blockchain or not. In order to introduce the product to the public, the firm publishes a white paper on its website, where all the technical and sometimes business information about the product and the future prospect of the project itself are provided. Based on this information, investors decide whether to purchase the tokens issued or not (Fisch, 2018). A token is a unit of value issued by a company and covers a wide range of applications. Usually, there are two types of tokens—utility and security, where the former is generally distinguished from the latter even though no legally binding classification of token types exists (Sameeh, 2018).

In fact, this all derives from the emergence of cryptocurrencies, which goes back to 2008, following the issuance of a Bitcoin white paper by Satoshi Nakamoto^4^. The emergence of cryptocurrencies led to the emergence of new financial assets like DeFi and cryptocurrencies where an ICO is one of these products. The actual hype around ICOs emerged after Ethereum entered the market with its new definition of money and built a platform that new startups could use to build their tokens and sell them on the market (Fisch, 2018). There are two ways through which ventures can issue tokens: either through their own developed distributed ledger technology (DLT)^5^ or through an existing DLT platform where they can develop their applications and use it as their own infrastructure (e.g., Ethereum, Waves—Sameeh, 2018). The most popular among these platforms is Ethereum. Ethereum-based tokens are known as ERC20^6^ or ERC223. Ethereum was the first platform to popularize and implement smart contracts and dApps (decentralized applications) which enable the use of Ethereum’s blockchain for various applications (Magas, 2018). Many experts also point out that this became a turning point for the early ventures in their bid to raise funds and finance early-stage development. However, there are also those who describe this as being only temporary due to the hype around cryptos and that these instruments will fade away in the near future. Unlike the previous critiques regarding ICOs, this field is still new and requires a lot of time to evolve and become a common way of raising funds.

### Initial Coin Offering Benefits and Drawbacks

Compared to similar ways of raising funds for SMEs, ICOs have pros and cons. On the benefits side, the most important features are as follows: disintermediation, network values, liquidity with time savings, inclusiveness, non-conferred ownership, and unlimited investor pool (ICO for SME financing, 2019). The disintermediation leads most of these companies to save on the costs of raising funds, which can be noticed in traditional ways of raising funds. Also, the company will have efficiency gains due to the use of blockchain and automation. The inclusive way of financing not only reflects the democratized way of raising funds but also the unconstrained access and active participation of investors. Additionally, there is an ability to invest in a certain portion of tokens and to quickly execute deals coupled with the near-intermediate liquidity of the market. There are usually many investors to these projects, who are very diverse and heterogeneous, as there is direct access to the global pool of investors (ICO for SME financing, 2019). In the case of ICOs, ownership is not conferred, meaning that the risk capital is raised without giving up ownership rights. Lastly, there is a valuable network that is being created where the customer base is formed and the value produced through the network’s effects.

In addition to crowdfunding, ICOs can be also compared to traditional ways of raising funds. For instance, an ICO resembles an initial public offering (IPO), as in both cases a venture issues digital tokens or shares to raise capital, which is then also traded on the secondary market (Ofir and Sadeh, 2018). One of the drawbacks is that in the case of ICOs, the venture does not undergo any specific requirements like estimating the value of a company through financial intermediaries or legal scrutinization, which are the backbone of traditional IPOs. Additionally, the ventures in the ICO case can be even at a concept level, whereas with IPOs, the companies are required to perform at least for a certain period of time and have viable financial data. In fact, these differences are avoided due to the disintermediation that takes place in all crypto products. However, this advantage is misused by some ventures, ones that raise significant amount of funds and then disappear from the markets by selling off all the tokens. These types of ICOs are known as scams where investors are exposed to fraud and lose their invested funds. According to Satis Group (Delisle, 2018), approximately 80% of ICOs in 2017 were identified as scams. Fortune Jack (Finance Monthly, 2018) found that just ten of the most high-profile ICO scams swindled $687.4 million from unsuspecting investors.

In our study, we use the data that were used previously by Fahlenbrach and Frattaroli (2020) in their study about successful investors, wherein they take into account only ICOs successful at that time. By successful, we mean ventures that have raised at least 90% of their hard capital^7^. However, among the projects that were accepted as successful before March 2018, almost 60% now have even 5% of amounts they raised (according to the coinmarketcap.com)—or they do not even exist anymore (based on their social media and news). Therefore, we have a clear case of ICOs which turned out to be scams after a longer period.

The main goal of our study is to identify the most important features, ones allowing us to identify scam ICOs ex-ante, as well as to discover additional insights for investors using machine learning techniques. We analyze a wide range of ICO characteristics and check which of them play an important role in scam ICOs. Our study is novel in the literature on the subject in three dimensions. First, we focus purely on the ICO features known in advance. Second, our definition of scams is wider and considers updated information not hitherto considered. Third, by using flexible machine learning algorithms, we can discover complex potentially nonlinear relationships and predict scams with greater accuracy. Finally, the application of XAI tools allows us to disclose the black-box models—i.e., to identify the most important features and uncover the shape of existing relationships between the outcome and individual predictors.

### Regulatory Uncertainty

A number of regulators have proposed differing approaches to the classification of ICOs. The Swiss Financial Market Supervisory Authority classifies tokens based on their underlying economic function into payment tokens, utility tokens, and asset tokens (FINMA Guidance, 2017). The difficulty in valuing ICO tokens is very much linked to the difficulty in defining tokens. If tokens were to be defined as a currency, their valuation would be similar to cash or cash alternatives; if defined based on their utility value, they would represent the price of the service at any point in time; if considered equity securities, the company’s enterprise value would need to be modeled and the price of the security derived from such model (ICO for SME financing, 2019).

In the case of ICOs, most of the tokens that are issued are either security tokens, utility tokens, or cryptocurrencies (i.e., payment tokens). In most cases, the companies that employ ICOs for their projects are avoiding supervision from the regulatory institutions by issuing tokens other than security ones (e.g., utility). The IPOs which pass the Howey test are identified as securities (Lyandres et al., 2019) and undergo the supervision from the regulatory institutions. According to the Howey test, financial products are identified as securities if a person invests their money in a common enterprise and is led to expect profits solely from the efforts of the promoter or a third party (Mendelson, 2019).

From the rational point of view, the safest type of tokens for investors is security tokens. However, this type of token makes ICOs more complicated and requires the application of know your customer (KYC) and anti-money laundering (AML) procedures (Myalo and Glukhov, 2019). Another level of difficulty in the valuation and pricing of ICOs relates to the way the value is created and shared within the network. Companies that are involved in ICOs should use blockchain technology since this creates the network effects which represent an important value creator for blockchain-enabled projects, thus the expected monetized value of such positive externalities needs to be accounted for in valuation (ICO for SME financing, 2019).

Nevertheless, when discussing the usefulness of ICOs, one notes the remarkable success of top projects, ones which in fact are still performing well today. One example is Chainlink, which held its ICO back in 2017 and still performs on a top level (i.e., disrupting the financial industry) and is the second among DeFi ventures based on market capitalization. However, in the case of tokens, it is challenging given the absence of any performance metric, the complexity in value creation and attribution in networks, and the difficulty in applying standard corporate finance theory on blockchain-based networks (Mendelson, 2019). Since this market is decentralized, we don’t know how and when proper regulations will come into force, but we know that many individual investors are devoting serious amounts of funds and are thus becoming prey for the vast amount of scam ICOs. Obviously, the pricing of the ICO projects is a big challenge for the industry nowadays, but with the available analytical tools, one can at least identify features that allow us to predict scams in advance, and this is the primary goal of our article.

### Predicting the Success of Initial Coin Offerings

Recently, there has been a lot of research related to the characteristics of the ICO market and the classification of ICOs based on differing sets of variables, where each article relied on a different methodology and arrived at insightful results. For instance, Stanley (2019) explored the application of behavioral heuristics like affect^8^ and representativeness^9^ to ICO valuation and investing. He considered six features as having an impact on investment decision-making due to behavioral biases—namely, the ease of understanding, coin value, market capitalization, maximum ICO bonus level, market sentiment, and pre-ICO social media levels. The Pearson’s correlation coefficient was used to analyze selected features against return on investment (ROI), assuming that successful ICOs have a higher ROI. The data covered the period before June 2018 was collected from numerous ICO platforms and websites. Fundamental analysis was taken from Coincheckup, and sentiment data were collected from Twitter. To utilize Twitter data, the Crimson Hexagon’s social sentiment analysis tool was used. The ease of understanding was calculated using Amazon’s web service template for evaluating the applicability of a blockchain project. The final dataset consisted of 47 ICOs in terms of ROI. The ease of understanding was found to be significantly correlated with ROI. Then the ease of understanding was combined with fundamental analysis to build a different model for evaluating cryptocurrency projects. As a result, this model outperformed the fundamental analysis alone, with a 33.6% improvement based on ROI. Based on this study, the current methods of fundamental analysis for blockchain projects are inadequate for capturing the full picture of the true potential of ICO projects. Investors with a lack of appropriate tools and constrained technical knowledge regarding the field of cryptocurrencies are influenced by behavioral factors.

Fisch (2018) introduced the ICO market to financial entrepreneurs by examining the factors that determine the amount of funding raised in ICOs. To explore this phenomenon, he referred to the signaling theory. This theory argues that high-quality ventures can attract higher amounts of funding by sending signals to potential investors (Spence, 1973). The projects utilizing DLT are technical in the sense that they are knowledge-intensive and technology-driven. Taking this into account, the author argued that the venture’s technological characteristics and capabilities are determinants of high quality and a prerequisite for success in a technological and innovation-driven industry. He derived three different indicators of a venture’s technological standing that should function as signals in the ICO context. In this study, the empirical dataset consists of 423 ICOs carried out between 2016 and 2018. According to the results of a multivariate regression model, the effective signaling of a venture’s technological capabilities is crucial for gaining higher amounts of capital. The technical white paper and high-quality source code lead to higher amounts of funding, but patents do not seem to have impact on the amount of funds raised. Additional features that play a role in the amount raised include token supply, usage of Ethereum standard, and Twitter activity. The results indicate that some of the underlying mechanisms in ICOs resemble those found in prior research into entrepreneurial finance, while others are unique to the ICO context. The results of the study are believed to raise awareness among investors, by helping them grasp the crucial factors impacting the amount of funds raised, as this fosters more informed decision-making, thereby reducing the considerable uncertainty faced when investing in ICOs.

Myalo and Glukhov (2019) discuss the formation of the model evaluation of ICO success. They apply a multivariate regression model based on data for 1,392 projects which were held in 2017 and the first half of 2018. As a dependent variable, they use three different features—namely, the amount of funds raised, the dummy variable indicating the success of the ICO (based on the (log) amount of funds raised: 1 if 100% or higher raised, 0 otherwise), and rating from the icobench.com (which is a platform that rates ICOs based on 20 different criteria). As independent variables, they use features from different groups, including ICO characteristics (e.g., duration of ICO, currency used, dummy variable explaining the existence of bonus), market characteristics (e.g., volatility data on Ethereum and Bitcoin), and rating characteristics (e.g., rating of the team, the product, vision of the project). The authors verify two research hypotheses: 1) the platform used for creating smart contracts matters to potential contributors and has an impact on the probability of a project’s success; 2) the transparency (availability and quality) of the information regarding ICOs impacts potential contributors and has a positive impact on the probability of a project’s success. As a result, the usage of the Ethereum platform (ERC-20) for smart contacts and dependence on the Ethereum volatility appear to be the most significant factors. Additionally, the findings indicate that the indicators of the sector of the project (almost all locations and regions) and the changing influence of the quality of the team are insignificant factors. This outcome may be explained by the lack of transparency, investor uncertainty about the venture (weak signals), and lack of the regulation and legal framework to control the market.

Furthermore, some of the scientific studies dive deeper into the topic and discover more patterns and dependencies using advanced statistical techniques like machine learning and deep learning. Toma and Cerchiello (2020) employ statistical approaches to detect what characteristics of ICOs are significantly related to fraudulent behavior. The manually built dataset is composed of 196 ICOs that occurred between October 2017 and November 2018 from sectors such as high-tech services, financial services, smart contracts, gambling platforms, marketplaces, and exchanges. The empirical data consist of ventures from several European countries—namely, Switzerland, Estonia, Latvia, Germany, Franceand non-European countries such as Russia, the United Kingdom, the United States, Japan, Singapore, and Australia. The authors consider both structured and non-structured data. The structured data were acquired from platforms such as icobench, coinschedule, and similar ones. In turn, the unstructured data consider white papers, websites, and messages from the Telegram platform. To turn the Telegram chats into numerical values, we use the Bag of Words^10^, Term Document Matrix^11^, and Term Frequency Inverse Document Frequency for weighting these matrices. The authors leverage several variables like entrepreneurial skills, Telegram chats, relative sentiment for each ICO, type of business, issuing country, and team characteristics. Classical statistical classification algorithms are applied to distinguish the status of the ICOs into scams and successful or failed ones. At the same time, they classify ICOs as failed or successful. A scam ICO is one which exhibited fraudulent activity with bad intent, as cited in the platforms above (icobench, etc.) or identified as such by any regulatory authorities (e.g., SEC) in having announced legal action against the venture. The successful ICO is one which collects predefined hard cap within the specified timeframe of the campaign, otherwise it is defined as a failed ICO. Through logistic regression, multinomial logistic regression, and text mining were used as a medium to reveal the features with highest impact in determining the success of the ICO. The results suggest that the presence of a website turns out to have a positive impact on the probability of not being a scam but does not have any impact on failed ICOs. Additionally, based on the sentiments expressed on Telegram chats, the impact appears to be negative both on the scam and failed ICOs. This suggests that monitoring Telegram chats in real time could provide a valid indication of possible problems within the ICOs. Finally, the presence of a Twitter account and white paper increases the probability of a ICO’s success.

Liu et al. (2019) overcome the challenge of measuring the fundamental trustworthiness of ICOs by constructing a novel measure from white papers. Using machine learning techniques, they construct a text-based Technology Index from a comprehensive sample of ICOs that took place from January 2017 to December 2018. The dataset consists of three different components: ICO characteristics from trackico.com, daily trading data from coinmarketcap.com, and textual measures from ICO white papers. The white paper in the ICOs is the most important source of information. Above all, it is the best source for deducing how much technology there is in a project. In this study, technology is a natural candidate for measuring the fundamentals of an ICO since all the projects relate to blockchain and also employ this technology most of the time. In particular, a machine learning method—word embedding—is used to capture the importance of technology in ICOs. The authors calculate the Tech Index based on industry-level statistics and data from GitHub. The final sample consists of 2,916 ICOs which together raised more than $17 billion. The dataset includes features as follows: ICO start and end date, total capital raised, trading status, platform, accepted currency, the founder team, industry, links of white paper, GitHub, and Twitter. In this study, there are two measures of success: 1) Trading, equal to 1 if tokens are traded in trackico.com and 0 otherwise; 2) Success, equal to 1 if the ICO has raised any capital and 0 otherwise. To deal with textual analysis, the authors use NLP techniques like bag of words (to capture semantics between words), word2vec (for vector representation of white papers), kNN (for classification), etc. Finally, they calculate the Tech Index which is the percentage of words in a white paper which belongs to a specific set of technological words. The authors first evaluate if Tech Index is related to ICO fund-raising and assume that if entrepreneurs cannot raise any funding, the ICO is not likely to succeed. Thus, one of the most important steps to ICO success is being capable of raising funds. The results show that the Tech Index offers a good proxy for the fundamental of an ICO and a good ICO should have a higher Tech Index. ICOs with a higher Tech Index are more likely to succeed and less likely to be delisted. Although the Tech Index does not affect short-run returns of ICOs, it has a positive impact on ICOs’ long-run performance. Overall, the results suggest that an important driving force is fundamental for the valuation of ICOs. What is more, ICOs with a higher Tech Index are more likely to raise capital and more likely to be traded in the secondary market subsequently. Thus, it appears that investors do care about the technology associated with ICOs. Furthermore, the authors investigate whether the technology aspect of ICOs is associated with its underpricing phenomenon. They find that the Tech Index is positively associated with ICO underpricing, indicating that the underpricing phenomenon is more severe for technologically advanced coins, which offers evidence that investors have difficulty in fully incorporating tech-related information, and thus undervalue the ICO with better underlying technology. This can be the consequence of the complexity of blockchain technology or stem from investor inattention.

Dio and Tam (2019) use NLP techniques (such as bag of words, counts, and TF-IDF, word sequence methods) and LSTM on two sources of data—white papers and websites—to classify the ICOs. The first neural network architecture focused on an ICO’s white paper with a bi-directional LSTM attention network. The second targeted the ICO’s website structure with a graph neural network as well as page topics with Latent Dirichlet Analysis. Finally, classification of the projects was based on various models such as max class, naïve Bayes, random forest, and logistic regression. The final dataset consisted of 1,975 websites and 1,023 white papers which took place between April 2013 and December 2018. The dependent variable takes three values: “failed”, “success”, and “risky”. Failed projects are those which raise less than 10% of hard cap, successful ones are those with more than 90% of raised capital, and the rest in between are counted as risky. The scores of white paper models (F1-score) as well as the method of how the information was taken perform more or less the same, where the bag of words (TF-IDF) technique appears to be performing a bit worse than bag of words (counts) and word sequence. Regarding the attention networks, sent2vec and word2vec do not differ with almost similar F1 scores. However, the recall in the word2vec case is 0.61 compared to sent2vec with only 0.53. In the website model, topics distribution is not enough to classify an ICO because the representation of the data is not distinct enough where 46% of the websites in data contain only a single page. Due to this, the model doesn’t pick up any significant correlation between ICO success and the topic in the websites. However, looking deeper into the classification, it is able to precisely specify the risky ICOs.

Bian et al. (2018) use a similar technique, but in a different way and on a wider dataset. The authors introduce ICORATING^12^, the learning-based cryptocurrency rating machine. They use NLP techniques to analyze various aspects of 2,251 digital currencies, using four groups of information such as white paper content, founding teams, Github repositories, and websites. The information from 1,317 white papers which is turned into texts using PDFminer API^13^, LDA for text clustering, and LSTM for mapping a white paper into a vector. Additionally, the authors use data about founding teams, dividing them into neural network features (features chosen using neural networks) and manually built ones. The Github README parts are handled as white papers, using an encoder-decoder model to map the file to a vector representation. Using Github, features like the number of branches, the number of commits, the total lines of code, and the total number of files are evaluated where the version before the time an ICO is used. The authors use the price change of an ICO project a year after the end of ICO itself as training signals. This price of 1 year after an ICO’s end is predicted using L2 distance between the predicted price change and the gold-standard price change. The ICO is identified as a scam if the predicted price is less than *m* percent of its ICO price. The amount of successful ICOs can differ for a different level of *m* (share of initial ICO price). According to the results, as the value of *m* increases, the proportion of the scam projects increases, leading to higher precision and lower recall. The features from the white paper and GitHub repository groups are the most important, giving F1 scores of about 0.7 when *m* is set to 0.1 and 0.5. Adding more features leads to progressively better precision and recall. After labelling 10 LDA topics and calculating the influence score of human-defined topics, ICOs on “gaming, gambling, and entertainment” are more likely to be scams than “exchange, payment, and smart contract”.

Based on the above review, we identify a research gap related to the extent of variables taken into account in classification of ICOs. Previous studies dealt with this issue from different approaches, analyzing the ICOs in a deep (Bian et al., 2018) or in a shallow way (Fisch, 2018). In our article, we approach this issue in a different way, using a vast number of features taken from different sources to analyze ICOs from various angles with special focus on those known in advance. Furthermore, in our study, scam ICOs are identified as those which are still active. This comes after noticing the many pump and dump schemes that took place on the ICO market between 2016–2018, when the token prices of those projects were decreasing dramatically in a matter of seconds and minutes, leaving investors with nothing. Additionally, there is a lack of thorough analysis of the shape of the relationship between the outcome (success or failure) and its predictors. For this purpose, we use XAI to uncover these relationships and understand how different characteristics of ICOs have an impact on its future success.

In this study, we test three research hypotheses as follows:

**First**, the characteristics of ICOs known ex-ante help to predict that an ICO is a scam. The dataset that we use (gathered from a range of sources) contains data for almost 300 ICOs with approximately 135 features (out of which 92 are known ex-ante). We state that the ex-ante features can help to predict scam ICOs with relatively high accuracy. The essence of ex-ante and ex-post features is also noticed in Roosenboom et al. (2020), where some ventures use fake ex-ante information for the sake of raising funds as well as good projects do.

**Second**, we state that the nonlinear machine algorithms allow us to predict the probability of a scam more accurately as compared to traditional logistic regression and its regularized extensions. These models may capture potentially existing highly nonlinear relationships between the outcome and its predictors.

**Third**, the technological background of ventures is an important factor determining the success of an ICO. The success of ICO ventures depends on their technological capabilities, where higher technological capabilities may correspond to higher quality in ICOs. Such ventures have a profound interest in signaling these capabilities to potential investors to obtain higher amounts of funding (Fisch, 2018). Adhami et al. (2018) find a significant relationship between the availability, even partial, of a project’s code with the probability of an ICO’s success. Also, the ventures with better technological background and capabilities are more likely to succeed and less likely to be delisted. This has a positive impact on ICOs’ long-run performance and valuation (Liu et al., 2019). In our case, we mainly focus on the following features: availability of Github (which has smart contract and project codes), the business model (e.g., availability of a white paper), if the venture is a decentralized platform, if the firm is a new blockchain company, and if the token issued is related to the blockchain or decentralization.

## Research Framework and Methodology

Multiple types of binary classification algorithms are applied. Logistic regression is used as the benchmark for other algorithms. The number of input features is large, therefore various methods of variable selection are applied. First, we use logistic regression with backward elimination based on AIC. Second, we apply simple filtering of predictors checking their one-to-one relationship with the outcome. Last but not least, we use machine learning models that automatically omit non-important, redundant features from the model (e.g., LASSO). In addition, one can expect that at least some predictors have a highly nonlinear impact on the probability of scamming and may interfere in complex interactions. To cope with these challenges, we apply several machine learning algorithms of various types. Apart from simple regularized extensions of the basic logistic regression (LASSO, ridge), we use another generalization of the quasi-linear approach—namely, the Support Vector Machine (SVM) with linear and polynomial kernels that allow us to better capture more complex nonlinear relationships assuming that the identified groups are well separated. In the end, we apply a group of homogeneous ensemble learners based on decision trees, both random forest as a representative of the bagging approach and several popular boosting models. The latter include extreme gradient boosting (xgboost), catboost, and lightgbm.

LASSO (Tibshirani, 1996) is applied as an efficient feature selection algorithm. It is an extension of a parametric regression model (here—logistic) that allows us to decrease the impact of less important features in the model and even to remove redundant predictors (set their parameters to 0).

The Support Vector Machine (Drucker et al., 1996; Vapnik, 1998) is another extension of the parametric model that uses a kernel trick to transform data into a more dimensional space, including nonlinear transformations of a feature space. We use polynomial and radial basis kernels to capture the nonlinear relationship of variables.

Random forest (Breiman, 2001) is one of the ensemble learning methods based on many tree models trained independently on bootstrap subsamples of original data. It takes into account only a random subset of features at each division.

XGBoost (Shapire and Freund, 1996) represents another family of tree-based ensemble learning–so called boosting algorithms that iteratively estimate subsequent models to gradually improve the model fit to the data. It uses a gradient boosting framework in a scalable and accurate way by pushing the computational power to the limits (Chen and Guestrin, 2016).

Catboost (Prokhorenkova, 2019) is a new gradient boosting toolkit with special techniques, the combination of which leads to outperforming comparable boosting implementations in terms of quality on a variety of datasets. Two critical algorithmic advances in Catboost are the implementation of ordered boosting and an innovative algorithm for processing categorical features.

Lightgbm (Ke, 2018) is a highly efficient gradient boosting decision tree (GBDT) where the authors use gradient-based one-side sampling (GOSS) and exclusive feature bundling (EFB). Lightgbm, a new way of implementing GBDT, speeds up the training process of conventional GBDT by up to over 20 times while achieving almost the same accuracy.

We divide our data into the training (70%) and test sample (30%) and perform hyperparameter tuning on the training sample using 10-fold cross validation. All models are estimated on two different sets of variables (explained with more details in the Data part): all potential predictors and a preselected subset of explanatory variables with relative stronger individual relationship with the outcome variable.

Most of the machine learning tools are “black boxes” which do not allow for easy interpretation of their results. However, various methods of explainable artificial intelligence (XAI) have recently been developed and can be used to explain or explore complex models. There are plenty of methods developed under the explainable artificial intelligence (XAI) umbrella that can be used to explain or explore complex models (Biecek and Burzykowski, 2021). Several machine learning algorithms offer model-specific measures for feature importance, but they cannot be directly compared between different model structures. Therefore, we use model agnostic methods which work independently on the structure of a model and are easily comparable. First, the model agnostic permutation-based feature importance metric will be used to identify important predictors. It calculates how the selected model accuracy measure changes if the values in a particular feature are randomly permuted. For an important variable, permutation breaks its links with the outcome and model will be less accurate. Second, we use partial dependence profiles that show how the expected model prediction changes with respect to the values of the selected explanatory variable keeping all other predictors constant (Kozak, 2018).

### Data

**FIGURE 2:**
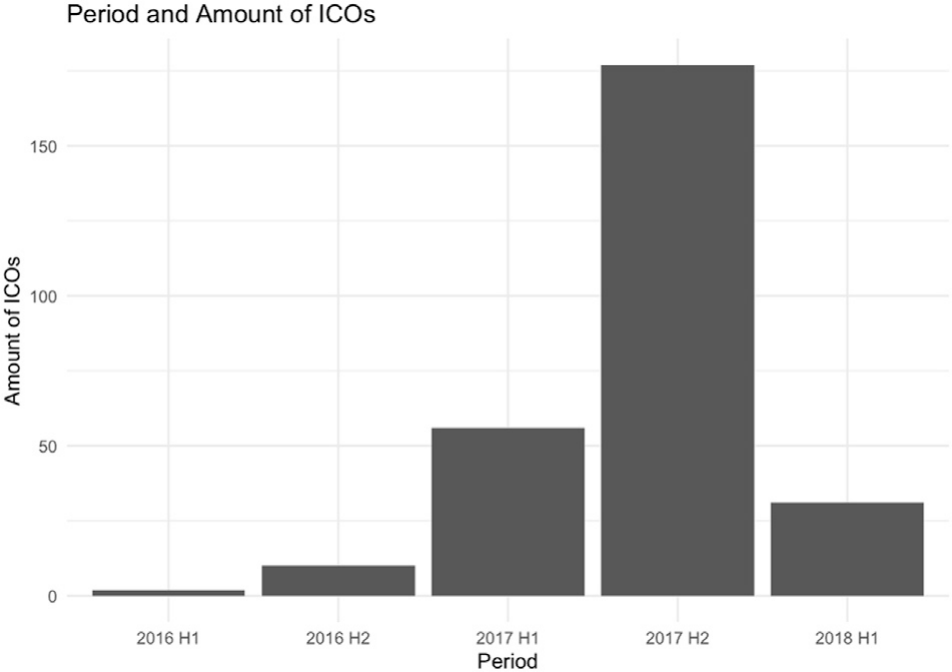
Number of ICOs from 2016 to the first half of 2018.

In this study, we use the structured data which was prepared and used by Fahlenbrach and Frattarolli (2020). The dataset contains all detailed information of 305 completed ICOs that took place from January 2016 to March 2018 when there was the first main hype around ICOs, especially during the second half of 2017 as can be seen in Figure 2.

The information about ICOs was collected from multiple sources–namely, company websites, white papers, social media, and Github pages where on average ICOs have 4,700 contributors (Fahlenbrach, Frattaroli, 2020). Websites like icorating.com, smithandcrown.com, icowatchlist.com, and coinschedule.com were used mainly to gather general information about projects, such as its name, start and end dates of the funding, the amount raised during these periods, the amounts of tokens issued, price of a token at the beginning and end of funding phases, the platform used, information about the team, information about the white paper, and social media channels.

**FIGURE 3:**
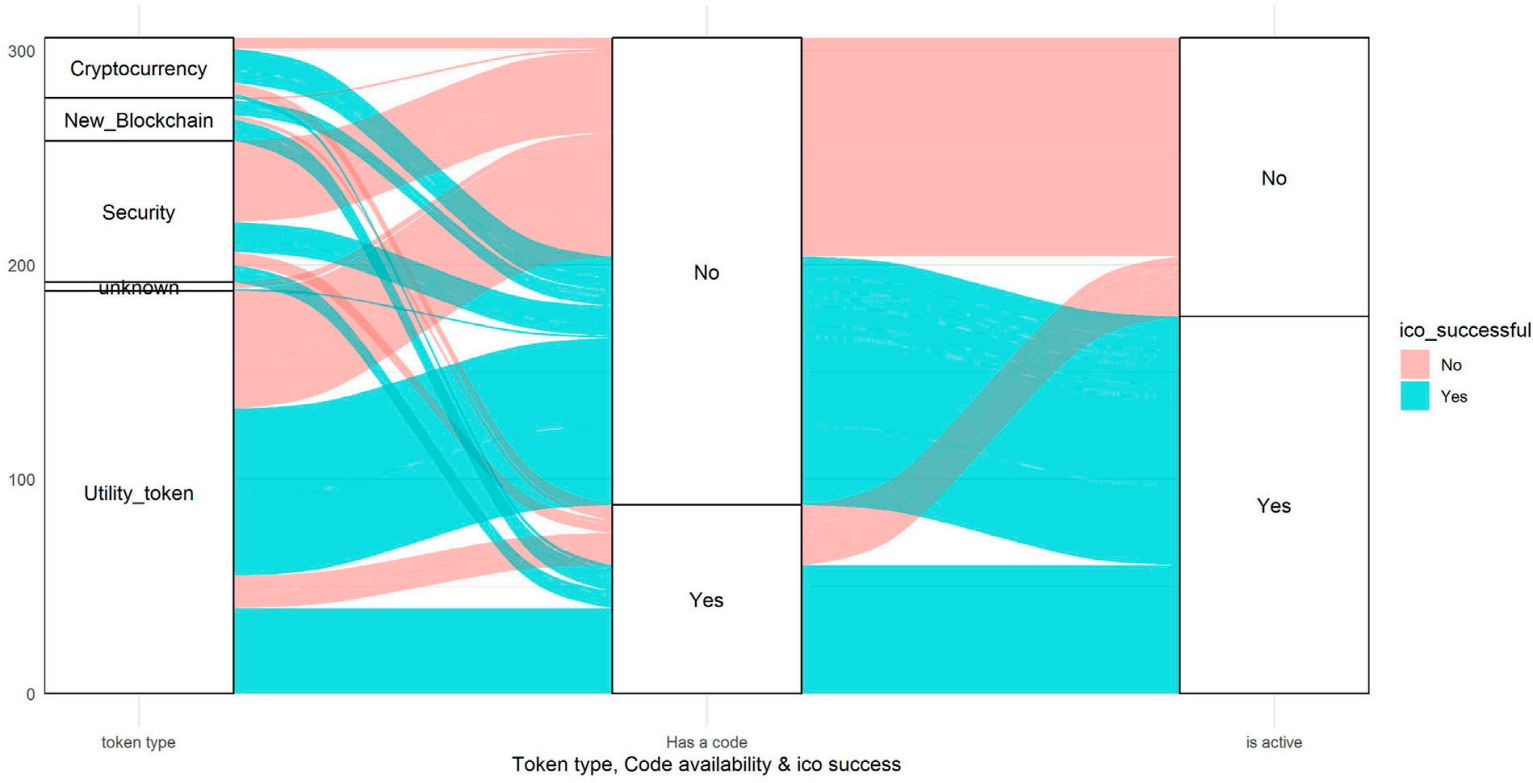
Token types.

Additionally, the dataset consists of dummy variables such as the availability of a website, white paper, and Github page. According to Fisch (2018), the more technical the paper the more funds can be raised, and that leads to a more successful ICO (Adhami et al., 2018). There are also categorical features related to the industry, token type, and token standard. In fact, most of the ICO tokens are of the utility type as this type can lead ventures to avoid regulatory constraints and issue tokens at any time without any supervision. Even if the venture issues the security type token, which is assumed to be safer since they undergo some regulatory procedures, most of these tokens turned out to be scams. Furthermore, one of the indicators of the technical ability of the venture—i.e., possession of a project code (e.g., Github)—does not indicate the success of the ICO where we see that almost half of those ventures with codes were scams as a result. However, having a project code is still better since most of the ventures without it would fail as a result. Overall, it is a bad idea to invest in ventures without project code selling utility tokens (Figure 3).

We furthermore have information about the legal aspect of the ICO, whether it takes appropriate compliance measures as know your customer and anti-money laundering or has any known legal form and jurisdiction. The information about the advisors and the team which are the backbone of the project is helpful in qualifying the ICO as more reliable where we mainly have the information about the number of advisors for each ICO and checking their educational background to assess the quality of the team. The remaining predictors are numerical with different scales.

In the case of ICOs, to assess a project’s business prospects and potential, one goes through the white paper, where one can find both technical information, business strategy, and some economic and financial results of the venture. To be successful in investing, one should not avoid synthesizing the business plan which might be a schematic and intuitive interpretation of the investment product. The feature that distinguishes a good plan is the clarity, the synthesis, and the professional description of the project workflow (Toma and Cerchiello, 2020). In our analysis, white papers served this purpose when we have features explaining its presence and length. Although a white paper normally may have a lot of information, the quality with which it is issued also plays a significant role, as the data contained within it and the description of the team’s components are crucial in every white paper.

In their study, Fahlenbrach and Frattaroli (2020) specify an ICO as a scam based on the amount of hard capital raised. Ventures that raised $1 million or more are mentioned as successful, others as a scam. Since these authors are interested in investigating the behavior of successful investors, they have chosen mostly the successful ventures. This in particular makes our case unique, since we know that almost half of these ventures which were successful at the time of raising funds are now scams.

We applied the updated classification of ICOs into two groups: 1) **scam**: an ICO which doesn’t exist anymore based on social media activities and delisting from listing platforms; 2) **non-scam**: an ICO which is still active in the market. The data are almost balanced—according to the above definition, we have 42.5% of scams and 57.5% of non-scams.

## Empirical Analysis

### Modelling Results

Before applying classification models, we performed feature engineering and initial feature selection to omit non-informative (e.g., near-zero variance) or redundant variables. All the initial data analyses and transformations were applied based on the distributions in the training sample, in order to avoid information leakage. In the case of binary variables (including one-hot encoded categorical predictors), we ignored those with a frequency of one of the levels below 5 (2.5% of the training sample). For three numeric features with right-skewed distribution, we applied the log transformation (log (x+1)) in the case of zeros among the values). Several variables were omitted due to a very strong relationship with other predictors (Pearson’s correlation above 0.95 for numeric variables, Cramer’s V above 0.95 for categorical predictors, or highly significant one-way ANOVA for categorical against numeric predictors). Token_share_producers_miners_ex_ante was omitted due to the large concentration of its values in 0. Finally, the full set of ex-ante features included 62 categorical and 12 numeric variables (labeled as “all” in tables with results). In addition, we applied simple variable filtering and selected features based on their relationship with the outcome. This subset included 20 categorical features (with Cramer’s V > 0.1) and seven numeric predictors (statistically significant at 10% level in one-way ANOVA)—it was labeled as “selected” in tables with results. Since the data are comprised of many binary features, for modelling purposes we applied range transformation for all variables, therefore all the predictors have the same range. Hyperparameter tuning was based on maximization of the F1 statistic (harmonic mean of recall and precision), but the assessment of all models was based also on other performance measures: area under ROC curve, accuracy, sensitivity (recall), specificity, precision, and balanced accuracy (average of sensitivity and specificity).

**FIGURE 4:**
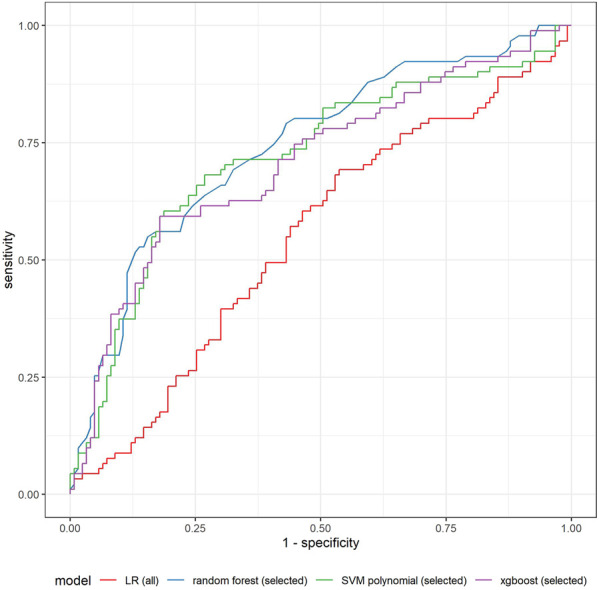
Comparison of ROC curves of selected models in the validation sample.

Table 1 shows the summary of performance of all models in the validation sample. One can see that ex-ante features allow us to distinguish between scams and non-scams with an accuracy up to 70%. Logistic regression (LR) does not provide superior performance (F1 equal to 0.47). Clearly, using only pre-selected features improves the model performance in all dimensions. The largest improvement is observed in ROC, precision, and F1 statistic. Adding backward elimination allows us to further improve the model based on all the features, but initial manual selection seems to work better. Regularized extensions of logistic regression (LASSO and ridge) produce higher F1 statistic, but prediction accuracy is highly imbalanced. The models are much better in predicting scams (very high sensitivity), while they lack accuracy in predicting non-scams (very low specificity, especially in the case of ridge). Better results are obtained with the application of another extension of the linear approach—support vector machine (SVM), especially with a polynomial kernel. Here one can clearly see the advantage of the initial pre-selection of variables. SVM with a polynomial kernel is more accurate than the variant using linear kernel function, which suggests nonlinearities between predictors and the outcome. In addition, the SVM model with a polynomial kernel estimated on selected variables consistently performs well independently of the measure applied. It has the highest balanced accuracy of all models (0.7041), second highest F1 statistic (0.6443), high accuracy (0.7190), and sensitivity (0.8038). Specificity, although lower than other statistics (0.6044), is still high among all the models applied. The remaining nonlinear algorithms are based on tree models (random forest, catboost, lightgbm, xgboost). These models are efficient in automated selection of the most important predictors. Therefore, when looking at their results, one cannot see such a strong advantage of using a limited number of predictors, apart from lower computational complexity. But the performance of these models on the dataset with selected predictors is usually better than when all the features are used. This is particularly evident in the case of the xgboost performance. The model estimated on selected predictors seems to be the best of all models estimated—its F1 statistic is the highest (0.6467), and it also has very high balanced accuracy. Random forest and catboost are much better in predicting scams (sensitivity above 0.8), but similarly as LASSO or ridge, they fail to predict non-scams equally well. Generally, in all cases, sensitivity is higher than specificity, which means that algorithms are better in detecting the scams than non-scams. But in the case of a xgboost on selected variables, the accuracy of predicting scams and non-scams has the best balance. All the above-mentioned measures depend on the selected cut-off-point. We used a default 50% as the distribution of scams and non-scams in our sample is almost balanced. Considering the area under the ROC curve, one can conclude that the random forest performs (slightly) better than the other models. When looking on the ROC curves of selected models (Figure 4), we can see that the curves for a random forest, xgboost, and SVM with polynomial kernel intersect with one another, and each of these models is clearly better than a traditional logistic regression. Taking all above discussed performance measures into account, we decided to take a more detailed look on the results of xgboost and a random forest, both based on selected predictors.

**TABLE 1:** Summary of models’ performance on a validation sample.

Model (variables set)	ROC	Accuracy	Sensitivity	Specificity	Precision	F1	Balanced accuracy
LR (all)	0.5592	0.5468	0.5859	0.4944	0.4711	0.4756	0.5402
LR (selected)	0.7044	0.6630	0.7071	0.6056	0.6251	0.6061	0.6563
LR + backward (all)	0.5914	0.5643	0.6006	0.5167	0.5086	0.4997	0.5587
LR + backward (selected)	0.6887	0.6394	0.6987	0.5600	0.5964	0.5705	0.6294
LASSO (all)	0.6805	0.6907	0.7955	0.5489	0.6693	0.5976	0.6722
LASSO (selected)	0.7270	0.6768	0.7308	0.6044	0.6401	0.6154	0.6676
ridge (all)	0.6501	0.6260	0.7147	0.5067	0.5767	0.5309	0.6107
ridge (selected)	0.7345	0.6911	0.7641	0.5933	0.6716	0.6221	0.6787
SVM linear (all)	0.6488	0.6108	0.6885	0.5056	0.5589	0.5246	0.5970
SVM linear (selected)	0.7204	0.7000	0.7795	0.5922	0.6730	0.6245	0.6859
SVM polynomial (all)	0.6488	0.6342	0.7045	0.5389	0.5981	0.5581	0.6217
SVM polynomial (selected)	0.7277	0.7190	0.8038	0.6044	0.7091	0.6443	0.7041
random forest (all)	0.7200	0.7050	0.8115	0.5611	0.7109	0.6212	0.6863
random forest (selected)	0.7458	0.7143	0.8276	0.5611	0.7238	0.6235	0.6943
xgboost (all)	0.6750	0.6775	0.7231	0.6178	0.6264	0.6158	0.6704
xgboost (selected)	0.7128	0.7093	0.7712	0.6256	0.6783	0.6467	0.6984
catboost (all)	0.7277	0.7195	0.8365	0.5600	0.7194	0.6243	0.6983
catboost (selected)	0.7335	0.7015	0.7808	0.5933	0.6652	0.6212	0.6871
lightgbm (all)	0.6788	0.6634	0.7333	0.5711	0.6065	0.5746	0.6522
lightgbm (selected)	0.7009	0.6686	0.7256	0.5911	0.6375	0.6036	0.6584

Therefore, the identification of the most important predictors was based on these models. Nonetheless, all the models were also compared on the test sample, which is summarized in Table 2.

**TABLE 2:** Summary of models’ performance on a test sample.

Model (variables set)	ROC	Accuracy	Sensitivity	Specificity	Precision	F1	Balanced accuracy
LR (all)	0.6575	0.6196	0.6792	0.5385	0.5526	0.5455	0.6089
LR (selected)	0.6971	0.6304	0.6415	0.6154	0.5581	0.5854	0.6284
LR + backward (all)	0.7199	0.6304	0.6604	0.5897	0.5610	0.5750	0.6251
LR + backward (selected)	0.7068	0.6630	0.7170	0.5897	0.6053	0.5974	0.6534
LASSO (all)	0.7131	0.6196	0.7358	0.4615	0.5625	0.5070	0.5987
LASSO (selected)	0.7059	0.6739	0.7547	0.5641	0.6286	0.5946	0.6594
ridge (all)	0.7068	0.6196	0.7170	0.4872	0.5588	0.5205	0.6021
ridge (selected)	0.7010	0.6739	0.7547	0.5641	0.6286	0.5946	0.6594
SVM linear (all)	0.6991	0.6739	0.7547	0.5641	0.6286	0.5946	0.6594
SVM linear (selected)	0.6831	0.6413	0.6981	0.5641	0.5789	0.5714	0.6311
SVM polynomial (all)	0.6986	0.6739	0.7547	0.5641	0.6286	0.5946	0.6594
SVM polynomial (selected)	0.6918	0.6522	0.7358	0.5385	0.6000	0.5676	0.6372
random forest (all)	0.6986	0.6522	0.7170	0.5641	0.5946	0.5789	0.6405
random forest (selected)	0.6606	0.6196	0.6604	0.5641	0.5500	0.5570	0.6122
xgboost (all)	0.6652	0.6087	0.6038	0.6154	0.5333	0.5714	0.6096
xgboost (selected)	0.6478	0.6413	0.6792	0.5897	0.5750	0.5823	0.6345
catboost (all)	0.6497	0.6196	0.6792	0.5385	0.5526	0.5455	0.6089
catboost (selected)	0.6023	0.5543	0.6226	0.4615	0.4737	0.4675	0.5421
lightgbm (all)	0.6584	0.6413	0.6415	0.6410	0.5682	0.6024	0.6413
lightgbm (selected)	0.6512	0.6522	0.6604	0.6422	0.5814	0.6098	0.6507

The results in test data show that by using previously selected models, one can still classify ICOs with an accuracy of 63–65%. Nonlinear models based on all features still have higher F1 and balanced accuracy statistics that the linear approaches, but the difference in performance measures is much lower. For LR, a substantial improvement in performance can be noticed. The LR estimated on selected variables consistently performs better for all performance measures considered. Adding backward elimination allows us to further improve ROC, specificity, and F1 statistic. LASSO and ridge regression have the most stable performance of all models as compared with the validation sample.

Again, the SVM model with a polynomial kernel estimated on selected variables consistently performs well on all measures applied except specificity. However, it does not perform as well as in the validation set. The remaining nonlinear algorithms are efficient in the automated selection of the most important predictors (they perform better on all variables than on a preselected group), except lightgbm, which shows the opposite. Overall, we observe the superiority of nonlinear models in identifying scam projects, especially in the case of validation set, used for model selection, where linear models are being substantially outperformed.

### Feature Importance

**FIGURE 5:**
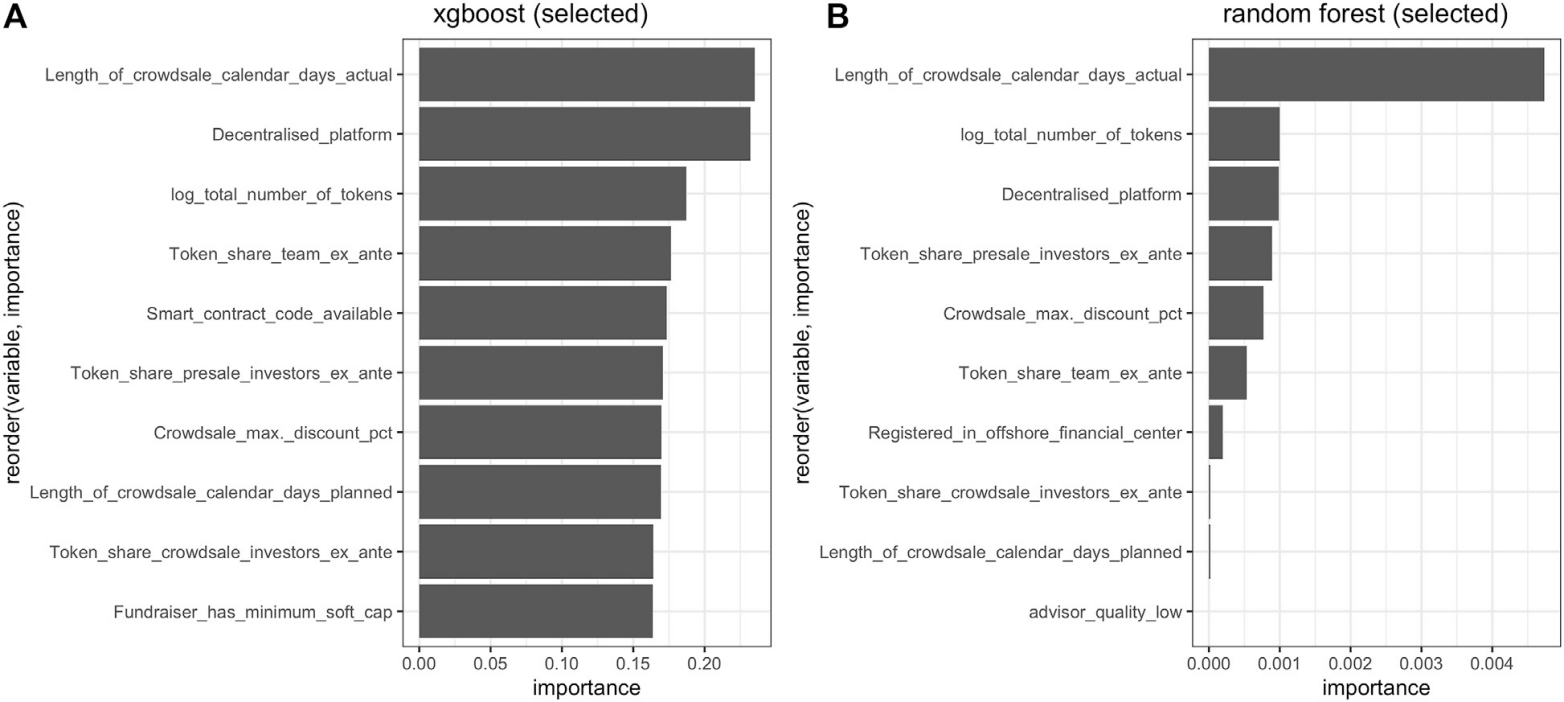
Feature importance for xgboost and random forest on selected variables (10 most important features).

To see which features play a crucial role in the success factor of an ICO, we evaluate the permutated feature importance for two selected models—an xgboost and a random forest (Figure 5). We measure the average increase in RMSE once a particular feature is randomly permutated. One can notice that 5 out of 6 top features in both models are identical with a slightly different order. The length of crowdsale calendar days, the total number of tokens, and the share of tokens offered ex-ante for the team and for investors in presales are crucial in determining the success of an ICO. The importance of the length of the crowdsale shows that the timeframe for gathering the funds as well as time provided to investors is critical. Investors also seem to care about their share in the project, which is important for the future decision in the whole venture’s activities. In addition, the use of a decentralized platform in the project and the availability of a smart contract code (in xgboost) are important determinants of scams. Additionally, being up-to-date in the technological sense is of crucial importance as well. The decentralization factor is important for a venture, especially the one that raises funds through ICO, where it creates additional value. Also, providing the smart contract code to the public shows the flexibility of the venture and that it is open for the further development, which often happens when they get updated on the Version Control Tools (VCT) such as Git or Gitlab. Furthermore, we notice the importance of the total number of tokens, providing a discount during the crowdsale and having minimum soft capital.

However, to identify the shape of the relationships of these features in a more detailed way, we investigate partial dependence plots (PDP).

**FIGURE 6:**
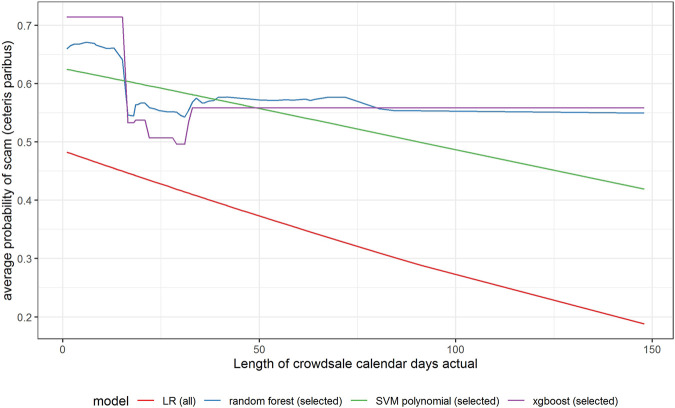
Partial dependence plot for variable indicating actual crowdsale calendar days.

**FIGURE 7:**
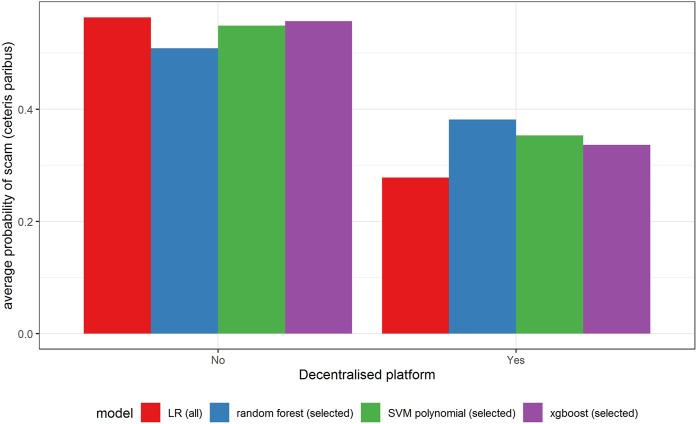
Partial dependence plot for variable indicating decentralized platform.

**FIGURE 8:**
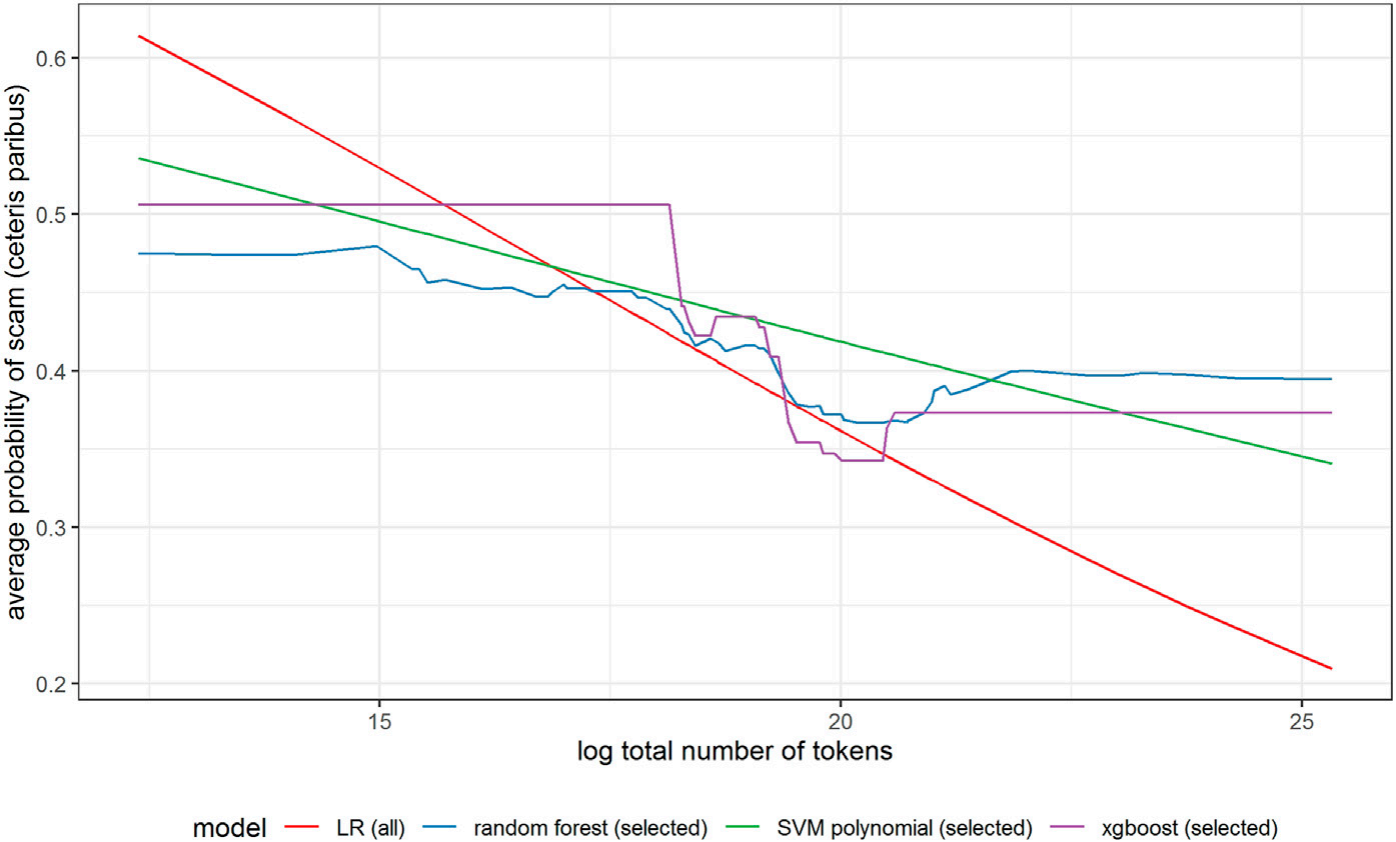
Partial dependence plot for variable indicating total number of tokens (log).

**FIGURE 9:**
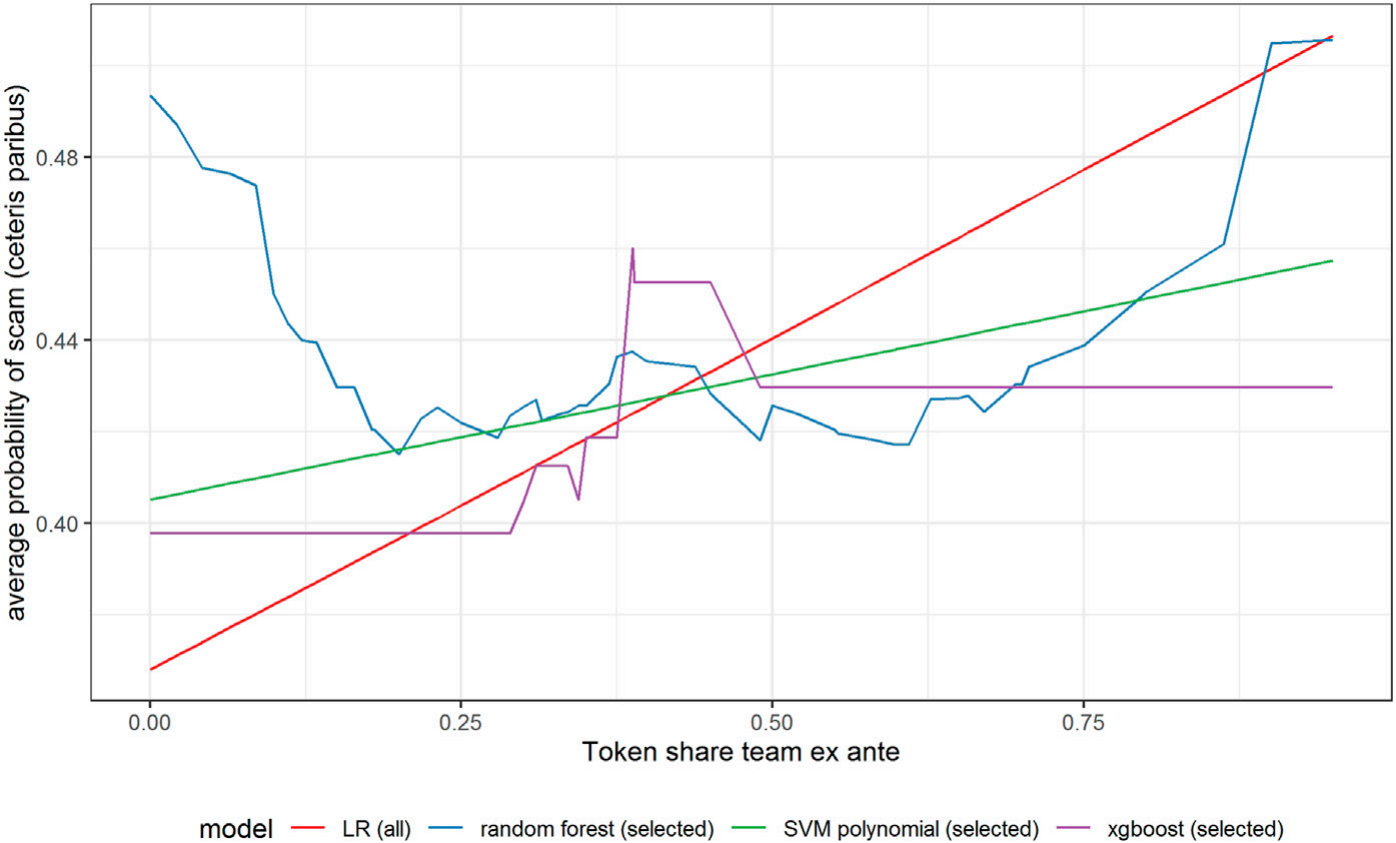
Partial dependence plot for variable indicating the share of tokens for the team (ex-ante).

**FIGURE 10:**
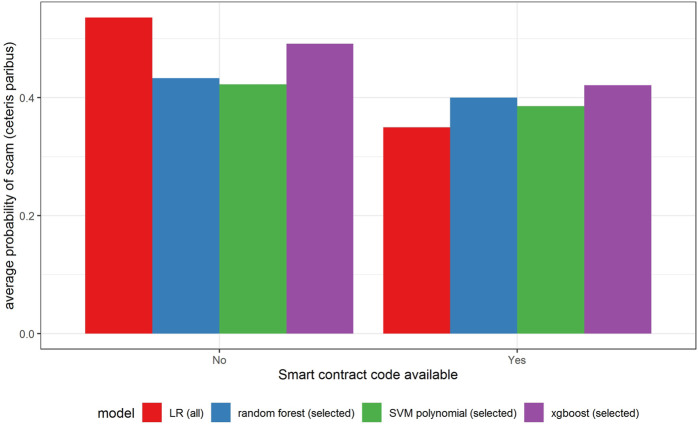
Partial dependence plot for variable indicating the availability of smart contract code.

**FIGURE 11:**
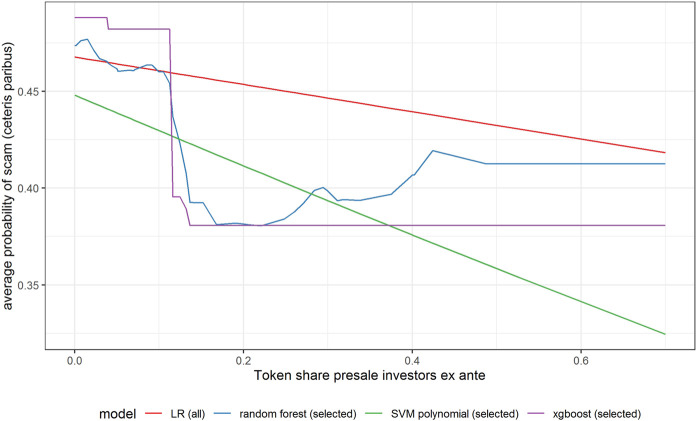
Partial dependence plot for variable indicating the share of tokens for the investors in presales (ex-ante).

In Figures 6–11 we evaluate the shape of the impact of the top 6 most important ex-ante features on an ICO’s success based on an xgboost and a random forest models together with two other models—SVM with a polynomial kernel, which performed relatively well in the validation sample and the linear benchmark—a logistic regression.

In Figure 6, we can notice that all models show a negative relationship between the planned length of crowdsale (days) and the probability of a scam. This means that the longer the crowdsale lasts, the lower the probability of a scam is. In other words, the longer an ICO is offered for investors, the safer is the investment. However, xgboost and random forest suggest that the negative relationship is not linear. The probability of an ICO being a scam is very high for very short offers (underestimated by LR); it sharply decreases if the crowdsale lasts more than 2 weeks, but it does not decrease further for offers lasting more than 1 month.

All models indicate that basing the offer on the decentralized platform (Figure 7) or the availability of the smart contract code (Figure 10) significantly lowers the probability of a scam. It also decreases with the number of tokens issued (Figure 8), but again the relationship is far from linear. An interesting relationship is visible for the share of tokens offered to the team (Figure 9). Logistic regression and SVM indicate a positive linear relationship, while a random forest suggests a U-shape curve, where the probability of a scam is relatively higher no matter if the team is offered a larger or smaller share in tokens. Xgboost in turn predicts that the risk of an ICO being a scam increases sharply to a higher constant level if the share of tokens obtained by the team is higher than 35–70%. A nonlinear relationship is also clearly indicated for the share of tokens offered to investors in presale (Figure 11). If this share is small (below 10%), the probability of a scam is relatively high, and it sharply decreases if investors are offered more than 10% of tokens. Xgboost suggests that a further increase in the share does not have any impact on the risk of a scam, while a random forest suggests that scams become more probable again if investors get more than 40% of the tokens in presales.

## Conclusion

ICOs may aptly be deemed one of the most controversial phenomena in the modern disruptive financial world. In the case of ICOs, being one of the easiest ways of crowdfunding through the usage of blockchain technology, this makes it vulnerable as a secure source of investment. Obviously, these projects lack transparency, technical understanding, and legality—and this leads to unscrupulous actors launching scam ICOs, something that spawns significant loss and places this infant world of cryptofinance in an unfavorable light.

In this study, we used machine learning to verify if a scam can be predicted based on features known in advance (ex-ante) and to identify the most important characteristics that lead to scam ICOs. By integrating different types of information about ICOs, using a wide range of variables showing various characteristics of the ICO market, the system is able to predict whether a project is a scam or not. We confirmed all three research hypotheses. The ex-ante characteristics of ICOs allow us to distinguish between scams and non-scams with a relatively high probability. Taking into consideration all performance measures, we confirmed the superiority, as compared to linear models, of nonlinear machine learning models in identifying warning signs hidden below the surface. However, this superiority was mainly visible in the validation sample and much weaker in the test data. Last but not least, we notice the importance of the technological capabilities of these ventures, which are a crucial factor in the future success of a project. Although it was not obvious that all technical aspects are important, we uncovered a positive relationship with the availability of smart contract code and being decentralized. The patterns and innovations revealed may help investors to identify reliable ICO projects and to make more rational decisions.

## Data Availability

The data analyzed in this study are subject to the following licenses/restrictions: data are supplied by Fahlenbrach and Frattarolli (2020). Requests to access these datasets should be directed to ruediger.fahlenbrach@epfl.ch.

## Footnotes

1 Decentralized Finance, ventures that are believed to disrupt the financial industry and replace traditional financial services.

2 Non-fungible tokens, cryptographic assets on blockchain with unique identification codes, and metadata that distinguish them from each other (https://www.investopedia.com/non-fungible-tokens-nft-5115211).

3 A white paper presents technical and business background information and the future prospects of the company.

4
Nakamoto (2008) published a study on Bitcoin and cryptography wherein he explained the digital currency.

5 Digital ledger technology is a system in which the transaction details are recorded in multiple places.

6 Ethereum request for comment, technical standard that is implemented by Ethereum.

7 The maximum amount of funds that the company targets to raise during an ICO.

8 The reliance on one’s intuitive judgment.

9 The assumption that individual characteristics represent the whole regardless of whether those characteristics actually relate to the whole.

10 In BoW, text is presented as an unordered collection of words.

11 For word and document vectorization.

12 The first learning-based cryptocurrency rating system was established by Shannon. AI.

13
https://github.com/euske/pdfminer
